# Fabrication of zinc oxide nanoparticles using *Ruellia tuberosa* leaf extract induces apoptosis through P53 and STAT3 signalling pathways in prostate cancer cells

**DOI:** 10.1515/biol-2025-1121

**Published:** 2025-07-11

**Authors:** Xianglin Guo, Haiyan Qu, Xiaoxu Lin, Zhengxiang Guo, Han Xia

**Affiliations:** Department of Urologic/Thyroid Surgery, Yantaishan Hospital, Yantai, Shandong, 264000, China; Department of Urology, Huaian Hospital of Huaian City, Huaian, Jiangsu, 223200, China; Hemodialysis Room, Yantaishan Hospital, Yantai, Shandong, 264000, China; Department of Nursing, Huaian Hospital of Huaian City, Huaian, Jiangsu, 223200, China; Department of Cardiovascular Medicine, The People’s Hospital of Dazu, Chongqing, 402360, China

**Keywords:** ZnO NPs, *R. tuberosa*, antioxidant, anticancer activity, P53/STAT3 pathway

## Abstract

The use of plant-based medicines for the production of green nanomaterials represents a viable route for cancer treatment. In this study, we report a novel method for the biosynthesis of zinc oxide nanoparticles (ZnO NPs) using the leaf extract of *Ruellia tuberosa* L. a medicinal plant known for its therapeutic properties. The study aims to test and investigate the ability of these *R. tuberosa*-derived ZnO NPs (RT-ZnO NPs) to cause apoptosis in human prostate cancer (PC) cells and clarify their fundamental molecular pathways. The developed RT-ZnO NPs were analysed employing field emission scanning electron microscopy, which exhibited a spherical shape with a median particle size of 156.7 nm. The methylthiazolyldiphenyl-tetrazolium bromide experiment showed that RT-ZnO NPs demonstrated considerable cytotoxicity toward DU 145 PC cells, with an IC₅₀ value of 26.82 µg/mL, while demonstrating low toxicity against normal HPE-15 prostate epithelial cells. Furthermore, the molecular analysis demonstrated that the NPs boosted p53 expression while suppressing both total and phosphorylated STAT3, indicating that anticancer activity is mediated by the p53 and STAT3 signalling pathways. This study focuses on a green and cost-effective method for creating anticancer nanomaterials, with RT-ZnO NPs emerging as a potential alternative for PC therapy.

## Graphical abstract

**Figure:**
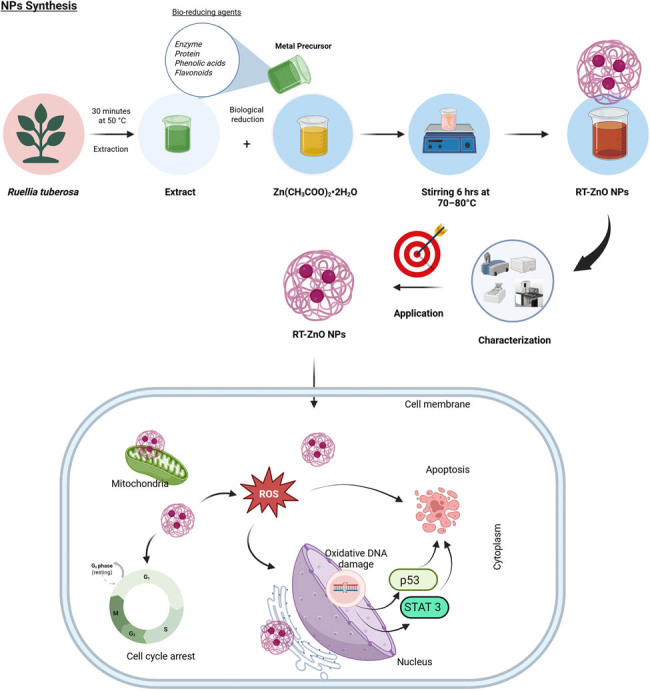

## 1 Introduction

Cancer is now the most prevalent cause of mortality worldwide, and its treatment costs are rising sharply as well. Prostate cancer (PC) has the highest incidence and mortality rate among all malignancies in males [1–3]. Approximately 20.3 million new instances of cancer and 10 million deaths from cancer were reported worldwide in 2022, according to recent data, and this number is predicted to increase in the years to come [4]. Even if there are simple methods for diagnosis and treatment, including chemically generated medications, chemotherapy, surgery, and radiation, patients still have severe side effects and stress as a result of these methods [5]. There is currently no treatment for some of the numerous malignancies that have been identified to stop their growth and progression [6]. Despite extensive study, cancer is still regarded as one of the largest health issues faced by human civilization. A major objective of the field’s investigation in malignancy therapy is to investigate and examine biological agents, including plant chemicals and herbal nanoparticles, due to the significant adverse reactions and resistance to drugs of chemical medications used in cancer treatment [7]. Thus, it is crucial to look for more potent medications made from plant chemicals and plant NPs that have fewer side effects and improved biocompatibility [8,9].

Nanotechnology, and especially nanomedicine, has transformed cancer detection and treatment because of its distinct physicochemical characteristics and molecular-level interactions with biological structures. Around 75–80% of the world’s population continues to employ traditional medical methods, stated to the World Health Organization [10], which emphasizes the need for plant-based advances in healthcare. Nanoparticles (NPs) are often created using both chemical and physical techniques. These days, researchers are increasingly more interested in the green synthesis of NPs since it avoids harmful chemicals, expensive materials, and difficult reactions [11]. The environmentally friendly production of NPs uses a variety of biological substances, including microbes and plants. Plants are thought to be the most promising natural resource for the production of metal NPs [12,13]. Among them, plant-mediated synthesis has drawn particular interest because of its ease of use, rapid development, and natural cap agents that support the stability and biological activity of the NPs [14,15].

The biological uses of zinc oxide (ZnO) NPs, such as their antibacterial capability, wound rehabilitation, antioxidant qualities, and, most importantly, cancer treatment, have been thoroughly investigated. The special physical and chemical physiognomies of ZnO NPs consist of retaining radiation, binding energy (60 meV), increased steadiness, a wide band gap (3.37 eV), electrochemical coupling coefficient, n-type semiconductivity, a high rate of chemical response in the presence of catalyst used, nontoxic, low-cost luminescent material, and so on [16,17]. To manage the particle morphologies and size distribution, several researchers began synthesizing ZnO NPs and modifying the existing ZnO NPs. Biomolecules, including polyphenols, flavonoids, amino acids, and organic acids, are abundant in plant extracts, which have two functions: they lower the metal ions and stabilize the resultant NPs [18–20]. These biologically active substances are essential for improving the biological and catalytic qualities of NPs in addition to aiding in their development and stability. By binding to the outer layer of the NPs, polyphenols, flavonoids, and other metabolites may behave as naturally occurring coatings that increase reaction surface area, target selectivity, and biocompatibility. By greatly influencing cellular absorption, reactive oxygen species (ROS) production, and enzyme-mimicking activity, this biological coating can increase the pharmaceutical and catalytic efficacy of the NPs [21,22].

This sustainable way of synthesis is capable of producing ZnO NPs with a variety of forms, sizes, and functional characteristics at low temperatures, despite the requirement for harmful chemicals or exogenous stabilizers [23]. As of now, metal oxide NPs have been synthesized using plants including *Ailanthus altissima* [24], *Costus woodsonii* [25], *Aquilegia pubiflora* [26], *Tagetes erecta* [27], *Trifolium pratense* [28], and *Morus rubra* [29].

This work is the first to provide data on the production of ZnO NPs utilizing leaf extract from *R. tuberosa*. *R. tuberosa*, sometimes referred to as Snapdragon root or Minnie root, is a member of the Acanthaceae family and is widely distributed across Asia. According to reports, *R. tuberosa* leaves contain anti-inflammatory, anti-microbial, and anticancer effects [30,31]. The primary objective of this investigation is to synthesize ZnO NPs using *R. tuberosa* leaf extract and evaluate their anticancer activity against PC cells, along with the underlying molecular mechanisms of action.

## 2 Materials and methods

### 2.1 Fresh sample collection and processing

After being thinly diced, 30 g of *R. tuberosa* leaves collected during the summer of 2023 in Chongqing, China, at the People’s Hospital of Dazu, were boiled for 30 min at 50°C in 100 mL of deionized water. A rotating evaporator device was used to reduce the extract’s volume. The resulting extract was then filtered, and the final powder obtained was 5 g. The ratio of fresh plant material to powder was approximately 6:1. The extract was stored at 4°C for further analysis.

### 2.2 Fabrication of *R. tuberosa* (RT)-ZnO NPs using *R. tuberosa* extract

Biogenic hydrothermal synthesis was used to create the NPs, following a method adapted from Rahaiee et al. [32] with slight modifications. Specifically, 0.878 g of zinc acetate dihydrate [Zn(CH₃COO)₂·2H_2_O, molar mass ≈ 219.5 g/mol] was dissolved in 400 mL of distilled water to achieve a 10 mM solution. To this, 80 mL of *R. tuberosa* leaf extract was added. To promote the development of NPs, 10 mL of 1 M NaOH was added to the reaction mixture to bring down its pH. The solution was then constantly agitated and kept between 80 and 80°C for 6 h. Following centrifugation of the colloidal mixture, the resultant NPs were dried for 24 h at 70–80°C in an oven.

### 2.3 Characterization of biogenic RT-ZnO NPs

The biogenically synthesized RT-ZnO NPs were characterized using various techniques such as Fourier transform infrared spectroscopy (FT-IR), scanning electron microscopy (SEM), energy dispersive X-ray (EDX) analysis, X-ray diffraction (XRD), dynamic light scattering (DLS), and zeta potential (ZP) analysis to determine their size, shape, and composition.

#### 2.3.1 FT-IR analysis

FT-IR (Bruker FT-IR Spectrometer) was used to evaluate the vibration patterns of the functional groups with activity that participated in stabilizing and decreasing the biogenetic RT-ZnO NPs on their surface. The FT-IR spectra of the R. tuberosa leaf extract and the synthesized nanoparticles were recorded using the potassium bromide (KBr) pellet method over the range of 4000–450 cm^−1^.

#### 2.3.2 Structural analysis

The structure of the biosynthesized RT-ZnO NPs was examined using FESEM. Elemental analysis was conducted using EDS with a Bruker XFlash 6I30.

#### 2.3.3 DLS and ZP analysis

DLS was used to measure the particle size distribution of the created nanocomposites (NCs), which were disseminated in Milli-Q water (1 mg/mL). ZP quantities were also performed to define the surface charge of the NCs.

#### 2.3.4 XRD analysis

The crystalline structure, purity, and size of the biosynthesized RT-ZnO NPs were examined using XRD. Cu Kα radiation (*λ* = 1.54184 Å) was used to study the XRD diffraction profile. 30 kV of voltage, 30 mA of electrical current, and examining across a 2*θ* angle range of 2–100° were used. Additionally, the Debye–Scherrer equation was used to determine the median crystallite size of ZnO NPs [33].

\[D\hspace{.25em}=\hspace{.25em}0.9xK/\cos \hspace{.25em}\theta ]\]

### 2.4 Cell culture and maintenance

The DU-145 PC cell line and HPE-15 human prostate epithelial cell line were obtained from Procell (Wuhan, China). DU-145 cells were cultured in RPMI-1640 medium, and HPE-15 cells were maintained in DMEM, each supplemented with 10% fetal bovine serum and 1% penicillin–streptomycin. The cells were incubated at 37°C in a humidified atmosphere containing 5% CO₂. All cell culture procedures were conducted under sterile conditions according to standard laboratory protocols.

### 2.5 Cell viability assay

The cytotoxicity of biosynthesized RT-ZnO NPs was assessed by the methylthiazolyldiphenyl-tetrazolium bromide (MTT) assay. Cells (1.0 × 10⁵ cells/mL) were planted in a 96-well plate, reared for 24 h, and then treated with different concentrations of the NCs (0–50 μg/mL) for an additional 24 h. Cell capability was evaluated by adding MTT and calculating absorbance at 570 nm. Cells were divided into three groups: untreated control, RT-ZnO NP-treated and 3.5 μg/mL of Docetaxel (DTX) [34] was used as a positive control for further experiments. DTX was used as a reference chemotherapeutic agent to evaluate the comparative anticancer potential of RT-ZnO NPs. The percentage viability was calculated using the following formula:

\[ \% \hspace{.5em}\text{viability}\hspace{.25em}=\hspace{.25em}(\text{Sample}/\text{Control})\hspace{.25em}\times \hspace{.25em}100]\]

### 2.6 ROS generation

The generation of ROS was investigated following established protocols [35]. Cells (5 × 10³/well) were cultured for 24 h, and then exposed to biosynthesized ZnO NPs for an additional 24 h. After treatment, the cells were rinsed with phosphate buffer solution and incubated for 30 min at 37°C with a 20 μM solution of 2′,7′-dichlorodihydrofluorescein diacetate (DCFH-DA). After removing the reaction mixture, 200 μL of PBS was added to each well, and flow cytometry was used to measure the amount of ROS generated.

For quantitative ROS measurement, cells (4 × 10³/well) were seeded in a 96-well black-bottom plate and allowed to adhere for 24 h. After exposure to biosynthesized ZnO NPs for 24 h, the culture medium was replaced with 20 μM DCFH-DA solution, and cells were incubated for 45 min at 37°C. The cells were washed with PBS, and ROS levels were measured using a microplate reader at 480 nm excitation and 520 nm emission wavelengths. Data were reported as fluorescence intensity relative to untreated control cells [36].

### 2.7 Oxidative stress induction

In cold water, the cells were broken apart using an ultrasonic cell crusher. The oxidation–antioxidation assay kits’ directions were followed when adding suspensions or supernatants to the reagents to determine malondialdehyde (MDA) and glutathione (GSH) levels and determining superoxide dismutase (SOD) and glutathione peroxidase (GSH-PX) activities. The Bradford test was used to measure the amount of protein present.

### 2.8 Dual staining assay

Apoptosis was assessed using 6-carboxyfluorescein diacetate (6-CFDA) and Annexin V-Cy3 staining. Cells were treated with biosynthesized ZnO NPs at IC_50_ concentrations, followed by staining and observation using a fluorescence microscope.

### 2.9 Evaluation of mitochondrial membrane potential impairment

After seeding DU 145 cells onto six-well plates, they were allowed to grow for 2–4 h under conventional culture circumstances. After that, the treated cells were incubated for 24 h under usual conditions with the addition of the IC_50_ concentration of biosynthesized ZnO NPs. Just 30 min before the incubation period ended, JC-1 stain (500 μL/tube) was given to the cells. The cells underwent two PBS washes after being centrifuged for 10 min at 1,000 rpm. Following the cells’ resuscitation in PBS, the BD-FACS Aria II flow cytometer was used to quantify the shift in membrane potential.

### 2.10 Analysing apoptotic cells using Annexin V/propidium iodide (PI) labelling

The efficacy of biosynthesized ZnO NPs to induce apoptosis was evaluated by flow-cytometry evaluation with Annexin V/PI stain, following a prior established approach [37] with slight alterations. To put it succinctly, IC_50_ doses of biosynthesized ZnO NPs were applied to 2 × 10^8^ cells/well, cultivated overnight. For 45 min the next day, cells were treated with a combination that included Annexin V/PI (500 μL/tube). With the use of a flow cytometer (BD LSRFortessaTM), 10,000 events/samples were then collected, and the FlowJo 10.8.1 program was employed to evaluate the results.

### 2.11 Evaluation of the cell cycle with PI staining

Employing the PI staining method, flow-cytometry analysis was carried out using a procedure mentioned earlier [38] with minor changes to evaluate the capacity of biosynthesized ZnO NPs to prevent the cell cycle during a single stage of its cycle. Briefly, IC_50_ doses of biosynthesized ZnO NPs were applied to 2 × 10^8^ cells/well after they had been grown for the whole night. Using 70% cooled ethanol (500 μL/tube), cells were held captive overnight at −20°C for the following day. After that, a solution containing PI (100 μg/mL), RNase A (10 μg/mL), and PBS (500 μL/tube) was added for 1 h. The data were then examined using FlowJo 10.8.1 Software after 10,000 events/samples were obtained using a flow-cytometer (BD LSRFortessaTM).

### 2.12 Western blot analysis

Western blot analysis was carried out to evaluate the expression of important regulation protein markers implicated in apoptosis. To put it succinctly, biosynthesized ZnO NPs were treated with 1 × 10^8^ cells/well and cultivated overnight. The cells were processed into lysates the next day. Following that, 50 μg of protein samples were separated by 10% SDS-PAGE and then put onto PVDF membranes. Following that, the membranes were incubated at 4°C for a whole night with certain primary antibodies. Following this, the membranes were exposed to the proper HRP-conjugated secondary antibodies for a length of 1 h. Following the detection of the signal utilizing the ECL Kit, the band pictures were taken using Image Lab 5.0 software. The Image J program was then used to quantify the band magnitudes, and β-actin was employed as the loading reference.

### 2.13 Statistical analysis

Data from the assays should be statistically analysed using GraphPad Prism version 9.1.0 (GraphPad Software, San Diego, CA, USA). Use one-way ANOVA or Student’s *t*-test to determine significant differences between groups, with *p* < 0.05 considered statistically significant.

## 3 Results

### 3.1 Physicochemical characterization and biosynthesis of RT-ZnO NPs

For the biosynthesis of RT-ZnO NPs, the leaf extract from *R. tuberosa* is a great and environmentally responsible option. By serving as a stabilizers and reducing substance, it has a dual function that improves the NPs’ effective synthesis. Initially, the colour shift of the solution to pale green and then off-white was utilized to visually check the generation of RT-ZnO NPs. The optical qualities and other features were then thoroughly examined using the following analyses.

#### 3.1.1 Optical characteristics

**Figure 1:**
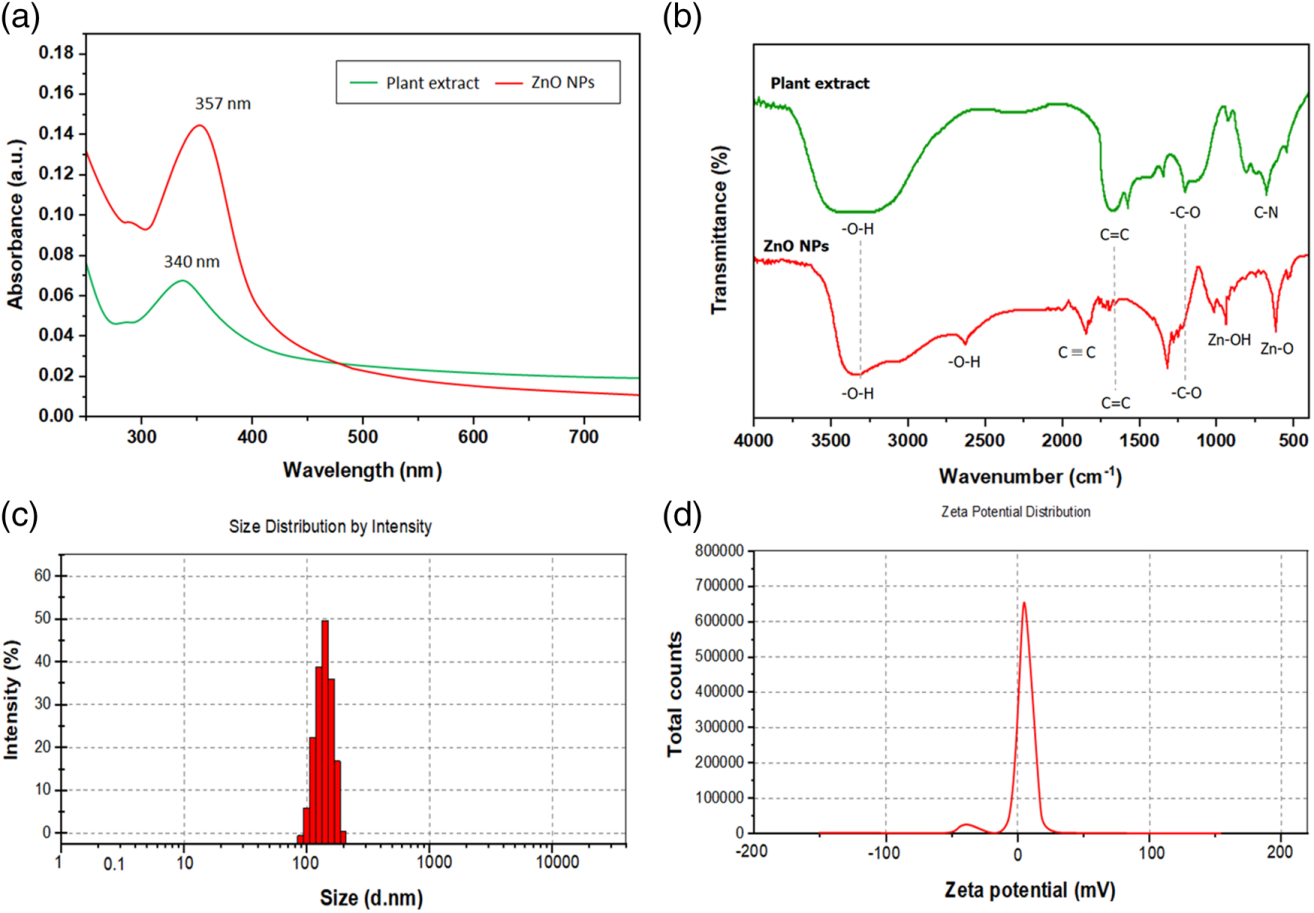
(a) Analysis of the UV–Vis spectrum of biogenically produced RT-ZnO NPs and *R. tuberosa* extract. (b) FT-IR spectrum of *R. tuberosa* extract and the biogenically synthesized RT-ZnO NPs. (c) Size distribution of the biogenically synthesized RT-ZnO NPs. (d) Zeta potential analysis of RT-ZnO NPs, indicating their stability in solution.

To verify the biogenesis of RT-ZnO NPs under ambient temperature in the wavelength range of 200–800 nm, UV–Vis spectroscopy was first performed. The characteristic peak absorbance measurement for RT-ZnO NPs was found to be at 357 nm, as shown by the UV–Vis absorption spectra, as seen in Figure 1a. ZnO NPs’ surface plasmon resonance (SPR), in which incoming light is absorbed due to the collectively oscillating pattern of unconstrained conduction band electrons, was the source behind this peak. The effective generation of pristine RT-ZnO NPs was additionally inveterate by the absence of any other peaks in the spectrum. Additionally, as shown in Figure 1a, the plant extract’s UV–Vis spectra revealed a captivation peak at 340 nm, suggesting that it consisted of phenolic substances.

#### 3.1.2 FT-IR analysis

As a result, FT-IR proved that phytocompounds were present on the outer layers of ZnO NPs. *R. tuberosa* leaf extract’s FT-IR spectra showed the existence of distinctive bands that corresponded to the functional groups of its phytochemicals, as seen in Figure 1b. Additionally, the characteristic bands of RT-ZnO NPs that were biosynthesized were examined among 4,000 and 450 cm^−1^. The obtained FT-IR spectra showed a substantial group around 3,400 cm^−1^, which is linked to the increasing resonance of the OH group in phenolic compounds. Two small peaks were subsequently seen at 2,920 and 2,850 cm⁻¹, correspondingly, which were used to identify the symmetrical and asymmetrical C–H stretching vibrations of the CH2 groups. Both spectra’s strong band at about 1,620 cm⁻¹ is attributed to carbonyl groups, or C═O stretching, which is frequently seen in flavonoids, tannins, and other phenolic compounds. The C═C stretching in aromatic compounds may be represented by the armpit at about 1,450 cm⁻¹. Additionally, O–H bending and C–N stretching may be the cause of the peaks at 1,380–1,310 cm⁻¹. Between 1,050 and 1,100 cm⁻¹, C–O and C–O–C stretching vibrations are visible. Then, three distinct bands were found at 700 and 610 cm^−1^, which point to Zn–OH and Zn–O bending vibrations that may be related to aromatic compounds. Finally, at 480 cm^−1^, the characteristic Zn–O stretching vibration mode was found. These results lend credence to phytocompounds’ dual function in ZnO NP fabrication as capping and reduction factors.

#### 3.1.3 RT-ZnO NPs produced via biosynthesis: Size and surface charge

RT-ZnO NPs’ hydrodynamic size and stability were determined using ZP measurement and DLS, respectively. Additionally, the ZP is utilized to determine the NPs’ surface charge and functionality. NPs’ varied applicability is ultimately determined by NPs stability, which is determined by the amount of their ZP. Particles with considerable stability are NPs with a high ZP. According to the findings of the study we performed, the particle size was 156.7 nm. The outcomes of the ZP study exhibited that RT-ZnO NPs had a polydispersity index (PDI) of 0.131 and a charge at 14.7 ± 0.134 mV (Figure 1c and d). The primary purpose of DLS analysis is to ascertain the particle sizes in various suspensions. The degree of particle aggregations in aqueous media was evaluated by the mean hydrodynamic particle diameter (*d*, nm).

#### 3.1.4 Characteristics of structure

**Figure 2:**
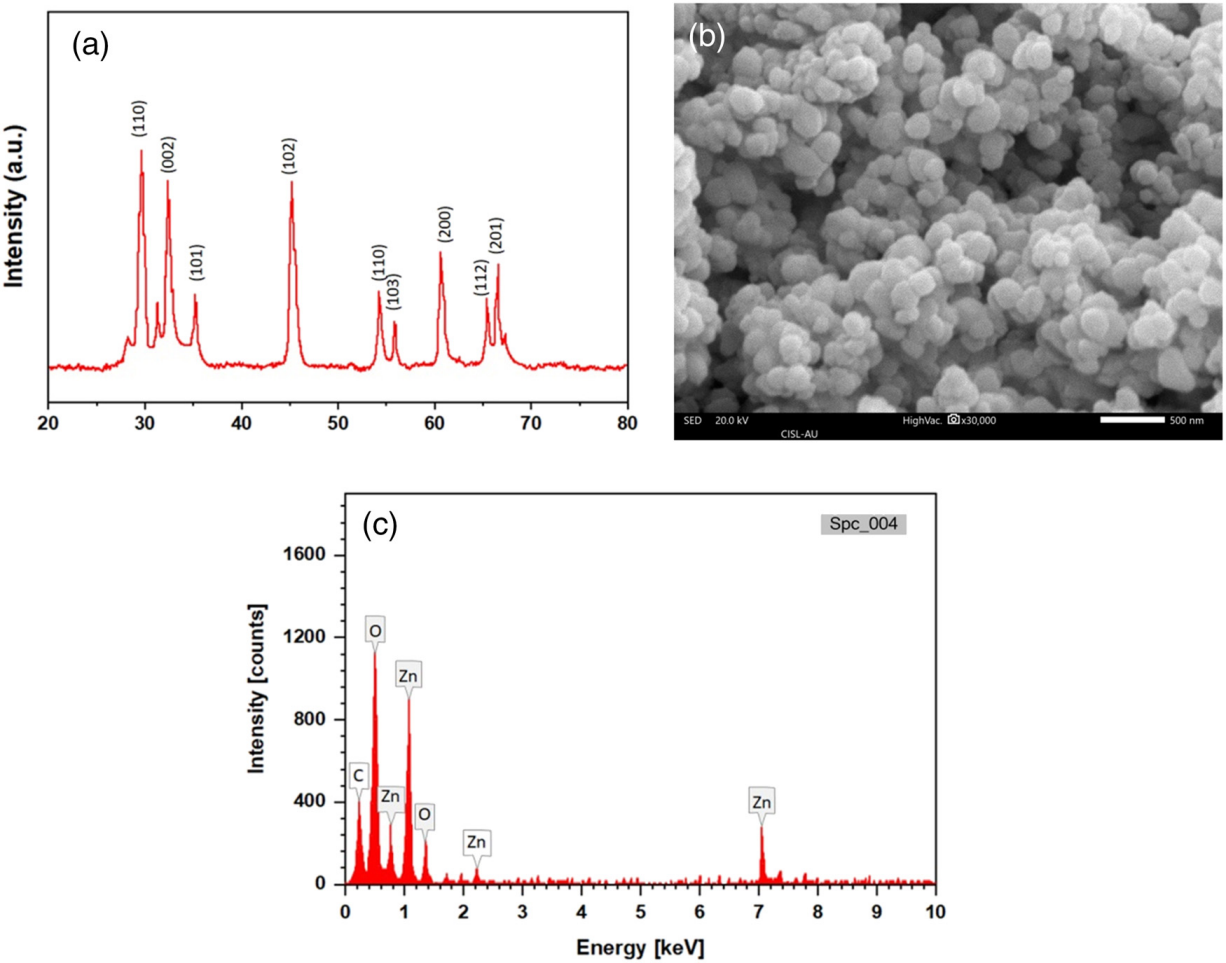
(a) XRD spectrum of RT-ZnO NPs in the 20–80° scattering range. (b) SEM analysis of biogenically synthesized RT-ZnO NPs. (c) EDX spectrum of RT-ZnO NPs, showing the elemental composition of the NPs.

By using XRD, the atomic structure formation, crystallization, as well as size of the produced NPs were correspondingly examined. The XRD form showed the diffraction peaks of the biogenically produced RT-ZnO NPs and respective Joint Committee on Powder Diffraction Standards (JCPDS) reference (JCPDS No. 89-1397), as shown in Figure 2a. With 2*θ* values of 29.89°, 33.42°, and 36.43°, the diffractogram first displayed three strong diffraction peaks that are ascribed to the (110), (002), and (101) crystallographic reflection planes. The 2*θ* values of 46.32°, 54.67°, 57.13°, 62.42°, 66.93°, and 67.95° were also found in additional prominent diffraction peaks. These correspond to the crystallographic reflection planes (102), (110), (103), (200), (112), and (201), respectively. The diffractogram displayed related degrees at 2*θ*, which are commonly seen in biogenic synthesis techniques and could be connected to the ingredients from the leaf extract surface crystallization on NPs. Additionally, the biosynthesized RT-ZnO NPs had a crystallinity percentage that was 88.12% and a normal crystalline size of 18.14 nm, according to the Debye–Scherrer equation.

#### 3.1.5 Compositional characteristics and structures of morphology

SEM was employed to observe the biogenic fabricated RT-ZnO NPs’ form and morphology. ZnO NPs have a mostly spherical or aggregated appearance; Figure 2b shows typical SEM photographs. EDX and SEM were used to determine the elemental structure of the generated RT-ZnO NPs (Figure 2b and c). The produced RT-ZnO NPs were mostly made up of Zn and O components, as demonstrated by the EDX spectra. However, oxygen showed a single peak close to 0.4 keV, while zinc showed four separate peaks at about 0.8, 1, 2.2, and 7.1 keV. These peaks align with the distinct features of ZnO NPs. The elemental analysis showed that the proportional percentages of O and Zn were 24 and 70.8%, respectively. The relative atomic percentages for Zn and O were determined to be 35.6 and 50.3%. At 0.26 keV, a low-intensity signal linked to carbon was also identified. The X-ray emissions linked to the leaf extract’s residual organic phytochemical components are responsible for this peak.

### 3.2 Assessment of the biosynthesized RT-ZnO NPs’ cytotoxicity toward PC cells

**Figure 3:**
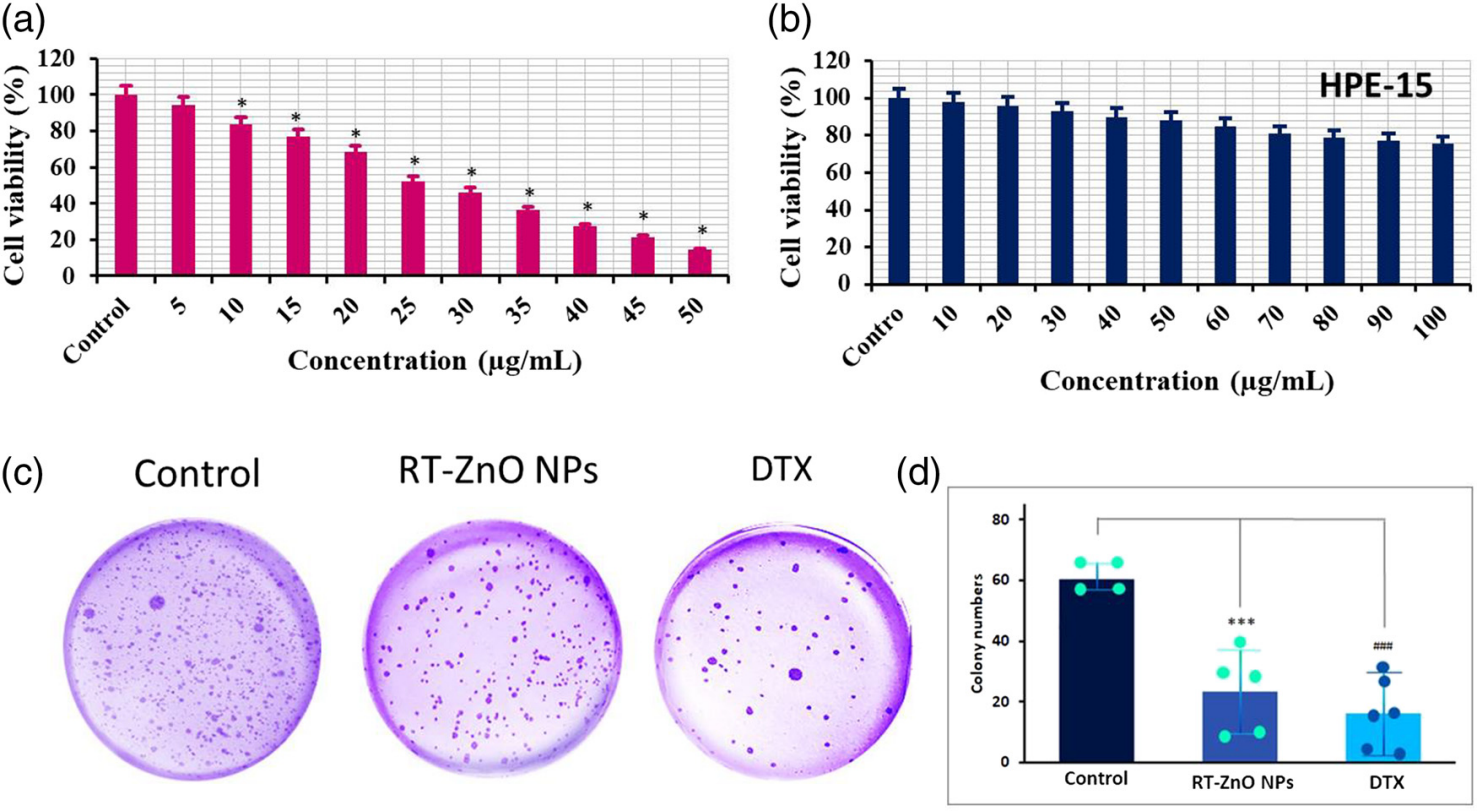
Anticancer activity of green-synthesized ZnO NPs by MTT assay. (a) MTT assay was used to assess the biosynthesized RT-ZnO NPs’ cytotoxic ability toward DU 145 cancer lines following the cells received pre-treatment with varying dosages of biosynthesized ZnO NPs for an entire night that ranged from 0 to 50 μg/mL. (b) Furthermore, it demonstrated nearly no cytotoxicity in normal prostate epithelial cells (HPE-15). The data are expressed in terms of mean ± SD (*n* = 3) *P < 0.0001. (c) and (d) Proportion of colony formation (%) after 24 h of treatment is shown in the bar graph demonstrating the clonogenic test of RT-ZnO NPs toward PC cells. The three replicas’ median and standard deviation are the statistics, and a distinction to the control sample is made at the ***P < 0.05 and ^###^ *P* < 0.01 significance levels.

MTT assay was used to assess the biosynthesized RT-ZnO NPs’ cytotoxic ability toward DU 145 cancer cells. Substantial concentration-related reduction of cell viabilities was then shown in the cell lines following the cells received pre-treatment with varying dosages of biosynthesized ZnO NPs for an entire night that ranged from 0 to 50 μg/mL (Figure 3a). According to DU 145 cell IC_50_ values, biosynthesized ZnO NPs had the most substantial cytotoxic capability against DU 145 cells (IC_50_ value: 26.82 μg/mL). Since DU 145 cells had an IC_50_ value of 26.82 μg/mL, all subsequent *in vitro* tests were conducted in these cells. Furthermore, it demonstrated nearly absolutely no cytotoxicity in normal prostate epithelial cells (HPE-15) (Figure 3b), suggesting that biosynthesized RT-ZnO NPs have little effect on normal cells at this dosage level in comparison to cancerous cells.

### 3.3 Biosynthesized ZnO NPs reveal an inhibition in DU 145 cell proliferation

Cancer cells’ capacity for independent survival and proliferation is determined by their capacity to form colonies. The clonogenic survival of biosynthesized RT-ZnO NPs against DU 145 cells was used to assess their anti-proliferative activity. Out of every picture, the control group showed full colony development, though the groups treated with biosynthesized RT-ZnO NPs showed a notable decrease in colony formation that was dependent on the IC_50_ concentration (Figure 3c). It was seen from the computed data (Figure 3d) that the IC_50_ values for biosynthesized RT-ZnO NPs steadily reduced the number of colonies in comparison to the control group. In DU 145 cells, the DTX treatment also drastically decreased colony formation. According to the results, DTX and biosynthesized RT-ZnO NPs were very comparable.

### 3.4 Biosynthesized RT-ZnO NPs induce oxidative stress in DU 145 cells

**Figure 4:**
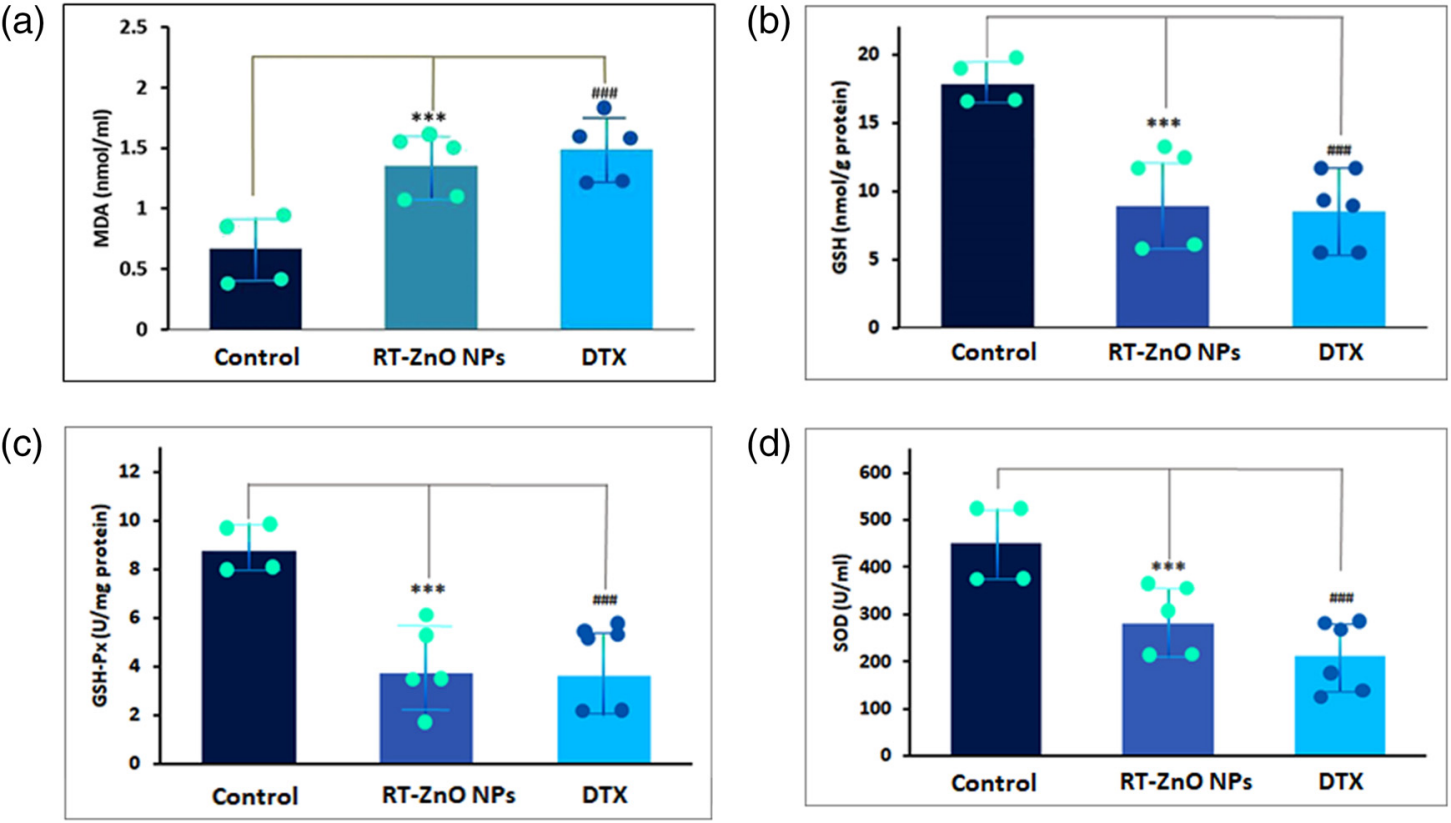
(a)–(d) RT-ZnO NPs induce oxidative stress in DU-145 cells. After treating the cells with RT-ZnO NPs for 24 h, measurements were taken for MDA content (a), GSH content (b), and the enzyme activities of GSH-PX (c) and SOD (d). The three replicas’ median and standard deviation are the statistics, and a distinction to the control sample is made at the ***P < 0.05 and ^###^ *P* < 0.01 significance levels.

Following a 24 h treatment with biosynthesized RT-ZnO NPs, the antioxidant enzyme activity of SOD and GSH-PX as well as the levels of MDA and GSH were assessed to determine if the NPs may cause oxidative stress in DU 145 cells. As seen in Figure 4, the biosynthesized ZnO NPs treated cells exhibited a large rise in MDA levels while exhibiting a considerable drop in GSH content and GSH-PX and SOD activities. These findings suggested that DU 145 cells might be exposed to oxidative stress as the outcome of biosynthesized ZnO NPs (Figure 4a–d).

### 3.5 ROS generation in DU 145 cells induced by biosynthesized RT-ZnO NPs

**Figure 5:**
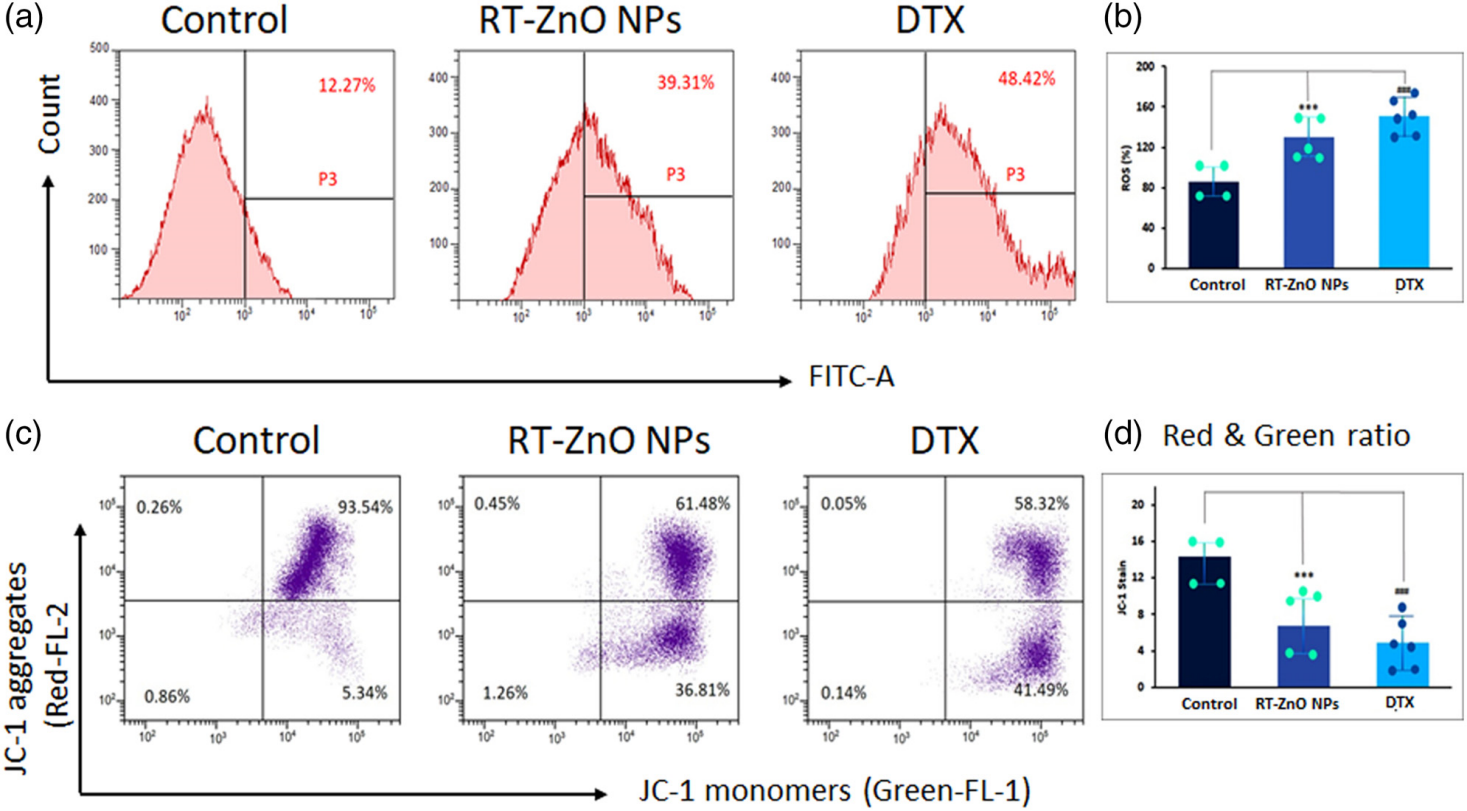
(a) and (b) Effect of green synthesized RT-ZnO NPs on the internal formation of ROS was investigated in DU-145 cells employing flow cytometric analysis of the dichlorofluorescein-stained fluorescence level. Observations from flow cytometry demonstrating how RT-ZnO NPs affect ROS generation. ROS generation was measured as a percentage using a spectrofluorometer. (c) and (d) The JC-1 quadrat plots illustrate the mitochondrial membrane potential, with JC-1 aggregates indicating mitochondria and JC-1 monomers representing the cytoplasm. The bar graph shows the ratio of JC-1 aggregates (red) to JC-1 monomers (green) following 24 h of RT-ZnO NPs treatment. The three replicas’ median and standard deviation are the statistics, and a distinction to the control sample is made at the ***P < 0.05 and ^###^ *P* < 0.01 significance levels.

The ability of biosynthesized RT-ZnO NPs to induce ROS intracellularly in DU 145 cells was assessed by employing the H_2_DCFDA dye. After entering the cells, the H_2_DCFDA dye was essentially deacetylated by cellular esterases to produce 2′,7′-dichlorodihydrofluorescein (H_2_DCF). This compound then interacted with ROS to produce DCF, which produced a bright green fluorescence. Groups treated with biosynthesized RT-ZnO NPs showed a notable determination by concentration production of intracellular ROS in spectrometric measurement data. The groups treated with biosynthesized RT-ZnO NPs had comparable results in the flow cytometry records (Figure 5a and b). Additionally, the microscopic examination confirmed the flow-cytometry findings. With increasing quantities of biosynthesized RT-ZnO NPs, it was evident that the cells’ green luminescence gradually enhanced when compared to a control. This suggests that biosynthesized RT-ZnO NPs have a substantial intracellular ROS activation capacity *in vitro*. Furthermore, the ROS levels in DU 145 cells were markedly increased by the DTX treatment. The findings revealed a striking resemblance between DTX and RT-ZnO NPs made by biogenic synthesis.

### 3.6 Induction of mitochondrial membrane potential loss (ΔΨM) by biosynthesized RT-ZnO NPs

The measurement of the proton difference through the innermost portion of the mitochondrial membrane, known as mitochondrial membrane potential (ΨM), is necessary for oxidative phosphorylation. Any change in the potential of the mitochondrial membrane serves as a signal for the unravelling of its inner membrane, which triggers apoptosis. The potential of the mitochondrial membrane is indicative of the functioning and stability of the mitochondria. Biosynthesized R-ZnO NPs were shown to produce ΔΨM in DU 145 cells using Rh-123 as a fluorescent probe. Utilizing flow cytometry, the change in Ψm was quantified. Exposure to the IC50 concentration of biosynthesized ZnO NPs led to a higher proportion of DU 145 cells undergoing apoptosis. The biosynthesized ZnO NPs thereby disturb the structure of the membrane and, in the case of the DU 145 prostate cancer cell, start the internal process of apoptosis (Figure 5c and d).

### 3.7 Pro-apoptotic effects of biosynthesized RT-ZnO NPs

The pro-apoptotic impact of biosynthesized RT-ZnO NPs was assessed using dual fluorescent labelling with 6-CFDA and Annexin V, as well as caspase-3 activity. 6. Phosphatidylserine externalization, a conventional indicator of apoptosis, is demonstrated by Annexin V, whereas CFDA measures cell viability. This means that just 6-CFDA (green) will show living cells; early apoptotic cells will also stain 6-CFDA and Annexin V (red); and late apoptotic cells will stain both Annexin V and DAPI (blue). The study’s findings suggest that biosynthesized RT-ZnO NPs have noticeably higher pro-apoptotic activity.

**Figure 6:**
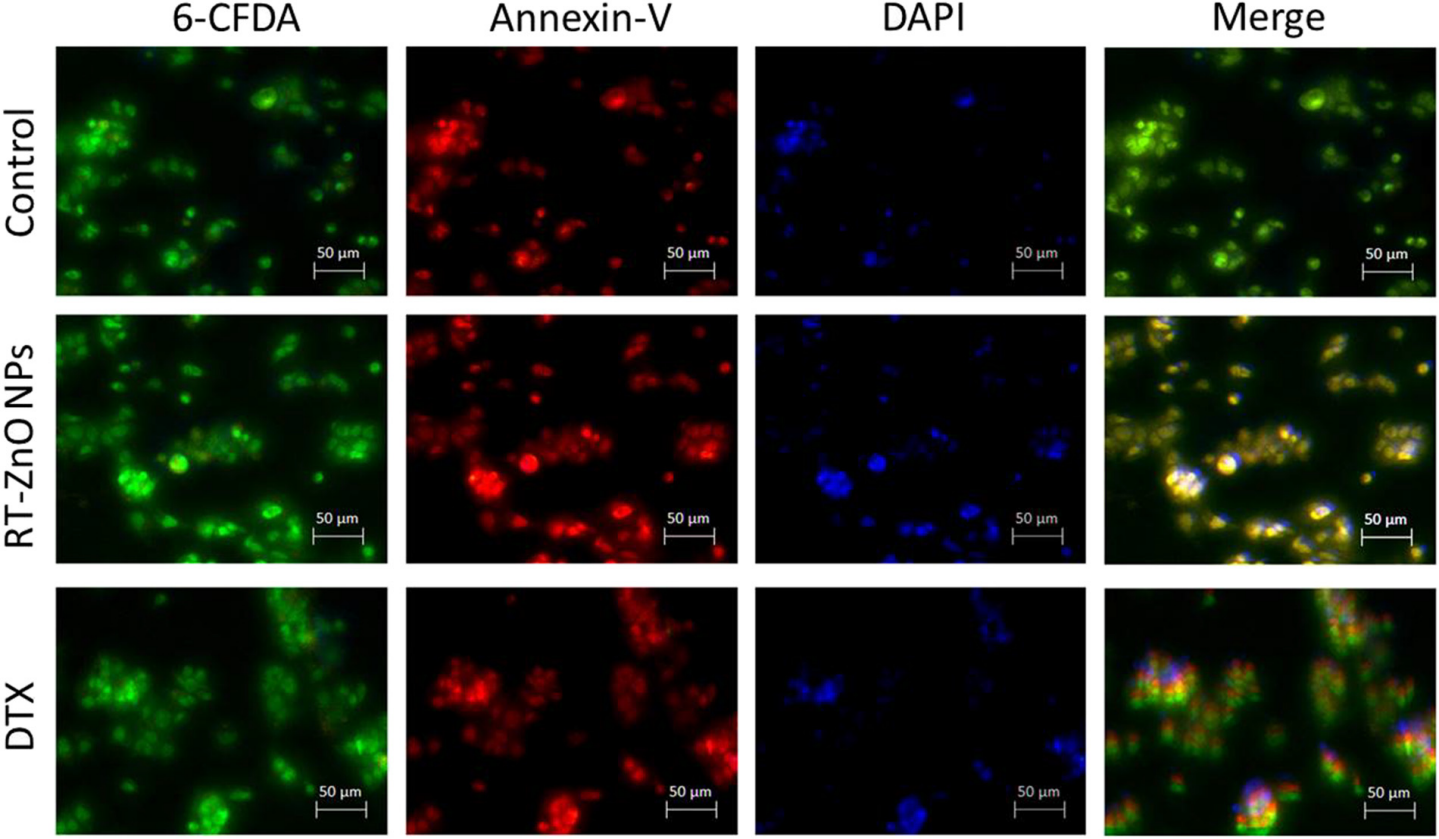
RT-ZnO NPs enhance pro-apoptotic effects. Fluorescent micrographs display DU-145 cells labelled with Annexin V (red), 6-CFDA (green), and DAPI (blue). Scale bar = 50 µm.

As seen in Figure 6, the percentage of Annexin V and 6-CFDA double-stained cells, in addition to Annexin V and DAPI-labelled cells, was increased in comparison to the control by the IC_50_ amount of biosynthesized ZnO NPs. Furthermore, when cells treated with biosynthesized ZnO NPs therapy were compared to treatments and control, they displayed significantly increased (*p* < 0.05) caspase-3 activity (Figure 6). A particularly significant pro-apoptotic elements is probably caspase-3, which is triggered by intrinsic and death ligand-mediated apoptosis processes. Biosynthesized RT-ZnO NPs made by DTX yielded comparable outcomes.

### 3.8 Biosynthesized RT-ZnO NPs reveal the G2/M cell cycle arrest in DU 145 cells

**Figure 7:**
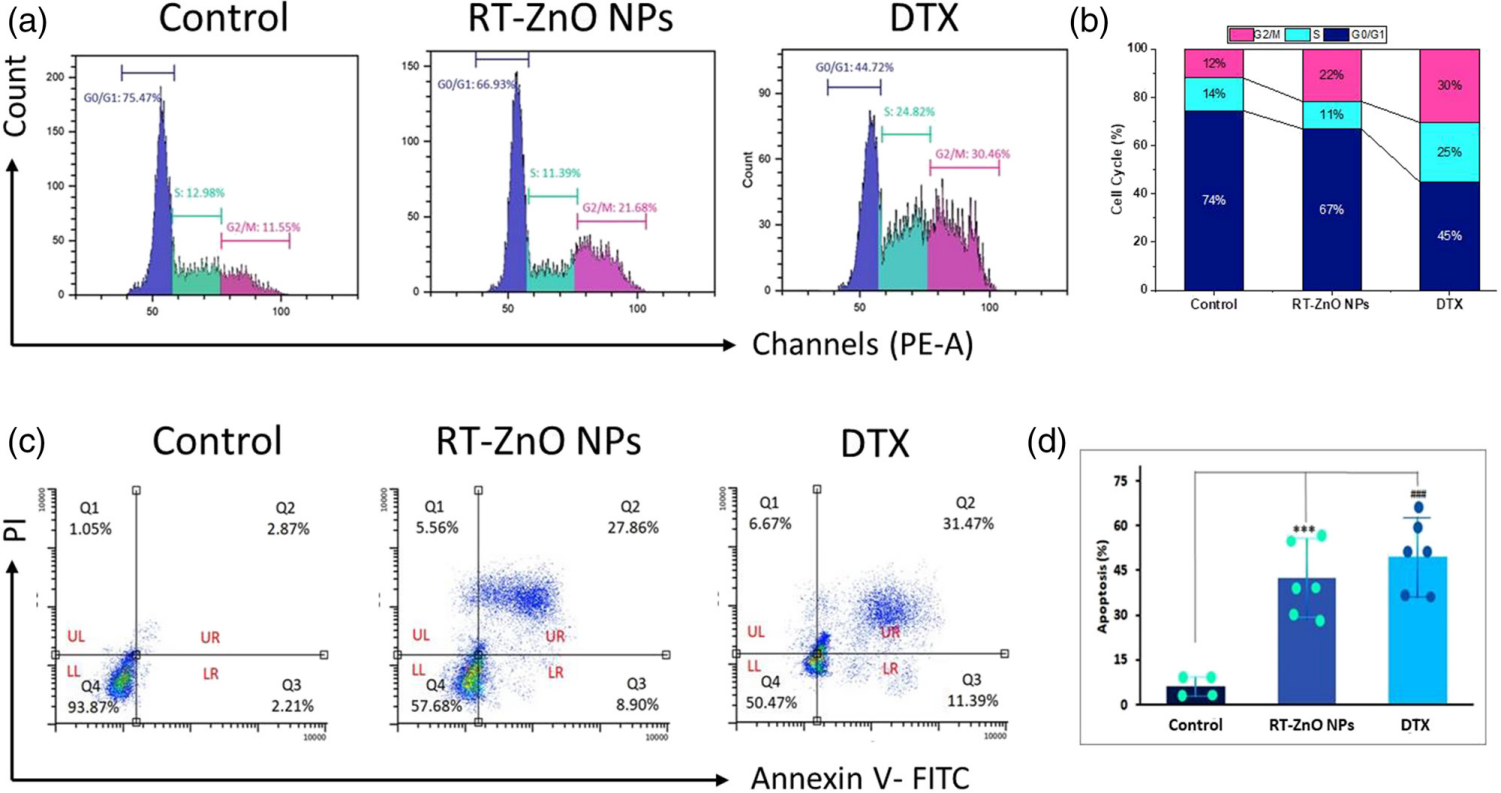
(a) and (b) Annexin V/PI quadrate plots display the percentage of apoptotic cells, while the bar graph indicates the total apoptosis rate (%) after 24 h of RT-ZnO NPs treatment. (c) and (d) Using flow cytometry, an examination of apoptosis was performed, and the results showed a significant difference concerning the control group of cells. The three replicas’ median and standard deviation are the statistics, and a distinction to the control sample is made at the ***P < 0.05 and ^###^ *P* < 0.01 significance levels.

The capacity of biosynthesized ZnO NPs to inhibit the cell cycle in DU 145 cells was assessed by flow cytometry analysis after PI labelling. Out of all the flow-cytometry histogram plots that were examined, the groups that were treated with biosynthesized ZnO NPs showed a notable G2/M stage of development arrest that was concentration-related (Figure 7a). With IC_50_ levels of biosynthesized RT-ZnO NPs, it was visible from the quantitative analysis (Figure 7b) that the G2/M populations progressively increased in comparison to the control group. This suggested that the accumulation of cells in the G2/M phase was the cause of the biosynthesized RT-ZnO NPs reliant on the arrest of cells. When compared with the control, the results of biosynthesized RT-ZnO NPs and DTX were practically the same. The results shown here demonstrate the strong anti-tumour effect of biosynthesized RT-ZnO NPs against PC.

### 3.9 Biosynthesized RT-ZnO NPs reveal the stimulation of apoptosis in DU 145 cells

The apoptosis-mediated response of biosynthesized ZnO NPs was confirmed by staining with Annexin V-FITC/PI labelling, and the results were measured by flow cytometry. DU 145 cells’ live and apoptotic cells were examined, as shown in Figure 7c. In the quadrate plotting of the groups treated with biosynthesized RT-ZnO NPs, the populations of Annexin V-FITC+/PI− (cells in early apoptosis) and Annexin V-FITC+/PI+ (cells in late apoptosis) gradually increased, but the numbers of Annexin VFITC−/PI− (normal cells) gradually decreased. It was clearly visible from the measurable flow-cytometry results (Figure 7d) that the overall apoptosis percentage demonstrated a significant reliance upon concentration. Stimulation of apoptosis in comparison to the control group. This suggests that biosynthesized RT-ZnO NPs have a significant *in vitro* capacity to prompt cellular apoptosis. In contrast, DU 145 cells treated with RT-ZnO NPs and DTX exhibited a much higher proportion of apoptosis. Nonetheless, the study demonstrated that the apoptotic efficiency of biogenically synthesized ZnO NPs against tumour cell lines was comparable to DTX results.

**Figure 8:**
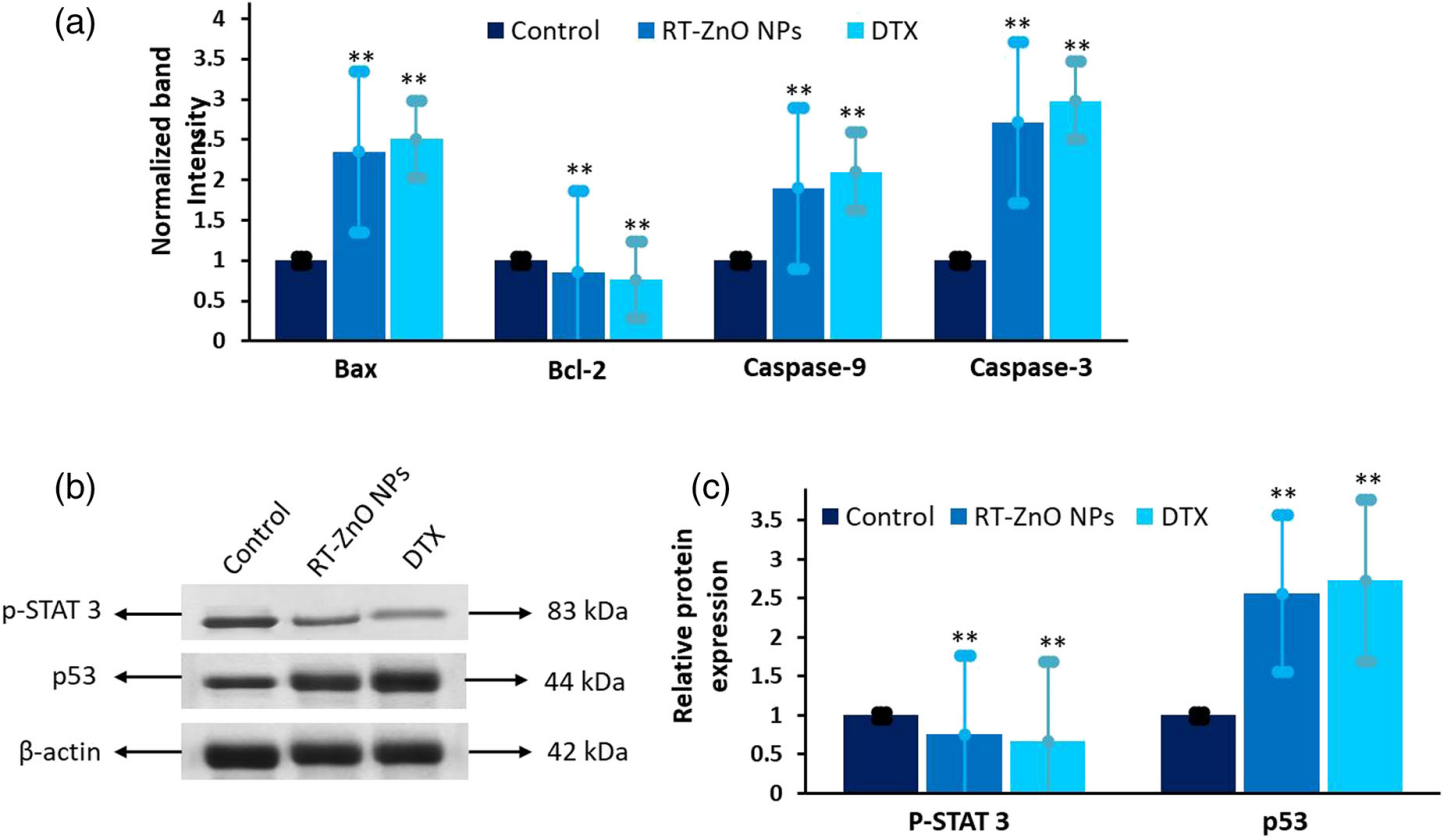
(a) Quantitative real-time PCR data were collected for apoptosis-related genes (Bcl-2, Bax, caspase 9, and caspase 3) and normalized to GAPDH. (b) and (c) Western blot data were collected for apoptosis pathway protein markers, including pSTAT3 and p53, with band intensities normalized to β-actin. The bar graph represents the relative intensities of pSTAT3 and p53 bands following 24 h of RT-ZnO NPs treatment. All experiments were conducted independently in triplicate, with data presented as mean ± SD. Statistical significance is indicated as **P < 0.01.

The appearance of caspase-9, 3, and Bax was significantly higher in cells exposed to biosynthesized ZnO NPs than in the control group, according to the findings of the RT-PCR test (Figure 8a). PC cells also showed decreased Bcl-2 expression. Cells treated with biosynthesized ZnO NPs showed a considerably increased Bax ratio. Furthermore, p-STAT-3 and p53 expression patterns were investigated. In comparison to the control group, it was shown that the administration of biosynthesized RT-ZnO NPs increased the pattern of p53 level while quashing the phosphorylation and expression of STAT3 in cells with PC (Figure 8b and c). As demonstrated by the elevation of pro-apoptotic markers (caspases and Bax) and a diminution of anti-apoptotic markers (Bcl-2), the treatment with an IC_50_ dose of biosynthesized RT-ZnO NPs often caused cell death. Additionally, biosynthesized RT-ZnO NPs enhanced p53 expression while inhibiting the production of both phosphorylated and total STAT3 in cancer cells, according to the molecular pathway study in biosynthesized RT-ZnO NPs cells. According to Figure 8b, this implies that STAT3 and p53 play a critical part in the therapeutic benefits of biosynthesized RT-ZnO NPs on prostate carcinoma cells.

## 4 Discussion

Nanoparticles may be made in a variety of ways, but biosynthesis is better than other processes since it is easy, inexpensive, and lacks harmful components and harmful organic solvents. The production of NPs using physicochemical processes necessitates excessive pressures and temperatures, costly and hazardous materials, and environmental harm. Sometimes, these harmful substances stay adsorbed on the NP surface, making it impossible to use the NPs in biomedical applications. In this work, *R. tuberosa* was used to produce RT-ZnO NPs. The leaves of *R. tuberosa* typically have a high concentration of polyphenol compounds, and are found in the secondary metabolic products of *R. tuberosa* extracts. These compounds have antimicrobial, antioxidant, anticancer, antinociceptive, and anti-inflammatory properties. The conversion of metal ions into metal NPs depends on the presence of polar soluble metabolites in plant extracts [30,31,38,39]. These biomolecules may contribute to ZnO NP reduction, stability, and capping.

ZnO NPs have already been made using a variety of medicinal plants [40,41]. In the current work, *R. tuberosa* leaf extract was used to create RT-ZnO NPs. The zinc acetate dehydrates and *R. tuberosa* leaf extract solvent mixture’s colour altered from pale green to half-white throughout the green synthesis of RT-ZnO NPs. The conversion of metal zinc (Zn^+^) ions to zinc (ZnO) NPs was shown by this colour shift. Physicochemical characterizations were performed on the resultant white material, which was presumed to be ZnO NPs. A UV–Vis spectrophotometer was used to measure the mixture’s UV absorption at wavelengths between 200 and 800 nm to track the reduction of zinc ions in the reaction solution. The generation of RT-ZnO NPs is indicated by the uptake band at 357 nm, which is associated with the SPR, as seen in Figure 1a. Furthermore, the abundance of phenolic chemicals and flavonoids, which are known to absorb substantially in this area due to π–π transitions in aromatic rings [42–44], is responsible for the noticeable absorption peak at 340 nm in the UV–Vis spectrum of the *R. tuberosa* extract. These metabolites likely played a dual role as both reducing and stabilizing agents during NPs synthesis. Employing EDX, the atomic content and composition of elements were verified. The synthesized ZnO NPs’ EDX spectrum is shown in Figure 2c, and the substantial signal verified that pure metallic ZnO NPs had been created. The signals associated with oxygen and carbon serve as protectors on the surface of ZnO NPs and could come from bioactive substances. The pure state of ZnO NPs was further confirmed by the observation of no further peaks associated with any components other than “Zn” and “O.” To assess the contribution of the biologically active substances in the aqueous *R. tuberosa* leaf extract to the creation of ZnO NPs, FT-IR studies were conducted. The identification of biomolecules in the field of natural substances is another use for the IR spectroscopic approach. As reducing and stabilizing agents, the existence of distinct IR bands shows diverse functional groups of biomolecules adsorbed onto the surface of ZnO NPs, confirming the biogenic synthesis of ZnO NPs (Figure 1b). Crucially, characteristic Zn–O vibrations were observed at 610 and 480 cm⁻¹, confirming NPs formation; C–O and C–O–C stretching vibrations appear near 1,050–1,100 cm⁻¹; a band at 1,620 cm^−1^ is associated with C═C, and a peak at 3,401 cm^−1^ is associated with OH stretching of alcohols and phenols. The peak forms show the presence of different biomolecules that have been found in earlier research. Previous studies have shown that Zn–O stretching vibrations are indicated by the IR bands that emerge around 470 and 800 cm^−1^ [45–48].

ZP also determines the surface charging and possible interactions of the produced NPs, and it is a key factor in determining the stability of the NPs and their consequent biological activities. The superior colloidal stability of NPs that have elevated ZP values (usually >±30 mV) is attributed to their higher electrostatic repulsion, which decreases agglomeration in dispersion [49]. The ZP of the RT-ZnO NPs in our investigation was +14.7 ± 1.134 mV (Figure 1d), suggesting a relatively stable behaviour that would be sufficient for short-term environmental or biological functions. Concerning negatively charged biological membranes, the positive charge is advantageous and may improve their effectiveness of cellular absorption and internalization [50].

A homogenous particle distribution with little aggregation was suggested by the DLS analysis, which showed an average hydrodynamic size of 156.7 nm and a PDI of 0.131 (Figure 1c). Because of their effective tissue penetration, extended systemic circulation, and increased permeability and retention effects in tumour tissues, NPs in the 100–200 nm range are typically thought to be ideal for therapeutic purposes [51], Our RT-ZnO NPs DLS findings are consistent with previous *R. virgata*-ZnO NPs findings [45]. The primary purpose of DLS analysis is to ascertain the particle sizes in various suspensions. The degree of particle aggregations in an aqueous medium was assessed by the mean hydrodynamic particle diameter (*d*, nm) [48,52].

SEM was used to examine the green synthesized RT-ZnO NPs’ structure and appearance. ZnO NPs have a primarily spherical form (Figure 2b). Green ZnO NPs were earlier produced from extracts of *R. virgata* and *G. wallichianum* by Iqbal et al. [52] and Abbasi et al. [53] with comparable outcomes. Additionally, XRD investigations were conducted to ascertain the crystalline nature and phase purity of ZnO NPs (Figure 2a). Using Scherrer’s equation, the crystal size of ZnO NPs was determined to be around 156 nm. ZnO NPs were previously created with *R. virgata, G. wallichianum*, and *Pelargonium odoratissimum* leaf extracts, with comparable outcomes documented [52,54].

Malignancy is the leading furthermost common cause of mortality globally. It is a malignant condition caused by unchecked cell multiplication. Of the many forms of cancer, PC is the second most often diagnosed disease in males, making up 15% of all male malignancies globally [55]. According to the most recent statistics, PC is the third most likely cause of tumour-associated deaths among males in the EU. With 78,800 fatalities expected in 2020, PC poses a significant health burden [56]. PC’s aetiology is still mostly a mystery. The potential for illness prevention is limited because there are now just a few known modifiable risk variables [57]. For individuals with progressive and/or metastatic illness, existing therapies are ineffective despite technical advancements [58]. More preclinical testing, investigation, and the creation of novel medications are urgently needed.

Creating novel anticancer medications with few adverse reactions and improved specificity and effectiveness is one of the most difficult areas of contemporary scientific study. The use of ethnomedicine dates back as far as human history, and multiple botanical extracts and plant-based complexes have been shown to possess antitumour and antioxidant qualities. Lem et al. [59], Guha et al. [60], and Seerangaraj et al. [30] reported a comparable cytotoxic activity of *R. tuberosa* extract against lung and breast cancer before the current findings. Additionally, phenols and polyphenolic chemicals were linked to cancer suppression, and lupeol and squalene might prevent breast carcinoma from spreading. Extracts from *R. tuberosa* also demonstrated anticancer effects on a variety of cancer cells [59,61]. However, numerous studies in nanomedicine have claimed that ZnO nanomaterials can be used to combat cancer cells, offering a potential target for the creation of anticancer mediators [62,63]. These findings corroborate our finding that biogenically synthesized ZnO NPs are significantly less harmful to healthy cells. The present results, that cytotoxicity varied according to the kind of cell and the substance employed, are completely consistent with other research [64]. But when cells were tested for ZnO NPs’ IC_50_ values, they were extremely toxic, exhibiting morphological abnormalities such as cell shrinkage and the complete collapse of their regular spindle structure. Comparable outcomes were seen for the cytotoxicity that biogenic RT-ZnO NPs caused in cancer cells after treatment. The plant-mediated production of ZnO NPs using extract of *Pandanus odorifer* showed the capacity to inhibit the development of different cancer cells, according to Hussain et al. [65]. Moreover, Mongy and Shalaby [66] found that the IC_50_ value of the green synthesized ZnO NPs using the *Rhus coriaria* for breast cancer cells [66]. Furthermore, the as-prepared ZnO NPs showed antitumour activity against breast cancer cells in the Stepankova et al. investigation [67]. Furthermore, the current findings supported the significant selective cytotoxicity of biosynthesized ZnO NPs to malignant cells that had been previously described.

The differences in redox states between malignant and normal cells may be the cause of the selective cytotoxicity seen, where RT-ZnO NPs significantly inhibited DU 145 carcinoma cells (IC_50_ = 26.82 μg/mL) with no harm to normal prostate cells. Because of their rapid metabolism and molecular instability, cancer cells usually have higher baseline levels of ROS, making them increasingly vulnerable to damage caused by oxidative stress [68,69]. Even while ROS are necessary for cellular communication and homeostasis, excessive production of these can be deadly, particularly in tumour cells with compromised antioxidant defenses [70,71]. We hypothesized a similar mechanism in DU 145 cells (Figure 4). It is probable that the RT-ZnO NPs specifically worsen oxidative stress in cancer cells, overpowering their weak antioxidant defences and causing apoptosis. Normal cells, on the other hand, have a stronger antioxidant system that can endure the ROS damage brought on by NPs. The ability of biogenic ZnO NPs to selectively activate oxidative stress-mediated pathways in tumour cells while preserving normal tissue can be explained by this mechanistic difference. ZnO NPs have shown comparable results in models of bladder and other PCs [72,73], and are consistent with the increased ROS and apoptosis markers observed in our current study. Intracellular ROS levels were assessed via the ROS-detecting fluorescent dye (DCF-DA) to validate our hypothesis. Following treatment with biosynthesized ZnO NPs, the amount of ROS was markedly elevated in a dose-related way (Figure 5). Once associated with the control set, ROS was dramatically generated in the present investigation, suggesting cellular death and cell cycle arrest.

To eradicate cells without endangering nearby cells, apoptosis, a predetermined sequence of actions that coordinates cell death, is essential [64]. Because the two primary goals of cancer treatment are to stimulate apoptosis and decrease metastasis [74]. It is essential for maintaining the balance between cell division and cell death, which supports healthy tissue homeostasis. Numerous NPs were seen to cause tumour cells to undergo apoptosis, which makes them potentially effective anticancer treatments [75]. To detect apoptosis-related morphological changes in DU 145 cancer cells following 24 h exposure to RT-ZnO NPs at IC_50_ concentrations, 6-CFDA and Annexin V-Cy3 staining were applied, targeting apoptosis-specific activity. As depicted in Figure 7, confocal microscopy revealed pronounced cell damage in DU 145 cells, characterized by a substantial loss of structural integrity, notable apoptotic features, and apoptotic body formation. To confirm the apoptotic action of biogenically synthesized RT-ZnO NPs, control and treated DU 145 cells have been stained with Annexin V-FITC/PI and analysed via flow cytometry. The distribution of viable and apoptotic cells is shown in Figure 6c. The present outcomes align with previous investigations demonstrating ZnO NP-induced apoptosis in numerous cell lines [52,66]. Following that, flow-cytometry analysis using JC-1 stain was used to quantify the modification of the membrane potential of mitochondria (MMP) that has a close connection with the basic process of cellular apoptosis [60]. The findings showed that biosynthesized RT-ZnO NPs substantially altered the MMP. Western blot analysis was thus used to evaluate the appearance of the mitochondrial critical regulating protein Cytochrome C to corroborate the MMP alteration findings. The current investigation exhibits that the expression of Cytochrome C was dramatically elevated by biosynthesized RT-ZnO NPs in comparison to the control group. Subsequently, western blot analysis was used to extensively examine apoptosis-related protein expressions to outline the molecular processes of biosynthesized RT-ZnO NPs. According to the densitometry study, pro-apoptotic Bax expression is dramatically elevated, though anti-apoptotic Bcl-2 expression is considerably reduced. Simultaneous starter caspase-9 and caspase-3 significantly increased in expression in caspase status, suggesting cellular death in DU 145 cells. Additionally, molecular pathway analysis in cells treated with biosynthesized RT-ZnO NPs showed that these NPs enhanced p53 expression while inhibiting phosphorylated and total STAT3 levels in cancer cells. This specifies that p53 and STAT3 show pivotal roles in the therapeutic influence of RT-ZnO NPs on PC cells.

## 5 Conclusion

The effective, sustainable synthesis of RT-ZnO NPs utilizing *R. tuberosa* leaf extract is demonstrated in the present study, providing a biocompatible and ecologically friendly substitute for NPs fabrication. The prospective use of RT-ZnO NPs for specific anticancer treatments was highlighted by their ability to specifically cause oxidative stress-mediated apoptosis in prostate tumour cells while sparing healthy cells. By combining phytochemical-rich plant substances for the production of NPs and demonstrating a direct mechanistic connection between ROS production, p53/STAT3 signalling regulation, and malignant cell death, this investigation is innovative. However, while these results indicate a correlation, further studies are required to elucidate the direct impact of RT-ZnO NPs on the activation or inhibition of the p53/STAT3 signalling pathway, These results demonstrate the potential of RT-ZnO NPs as a potential option for targeted PC therapy, with minimal effects on healthy cells, highlighting their usefulness in cancer nanomedicine and the development of environmentally friendly therapeutics.
